# Engineering of Methylation State Specific 3xMBT Domain Using ELISA Screening

**DOI:** 10.1371/journal.pone.0154207

**Published:** 2016-04-25

**Authors:** Dan Od Cohen, Shai Duchin, Michal Feldman, Raz Zarivach, Amir Aharoni, Dan Levy

**Affiliations:** 1 The Shraga Segal Department of Microbiology, Immunology and Genetics, Ben-Gurion University of the Negev, Be’er Sheva, Israel; 2 Department of Life Sciences, Ben-Gurion University of the Negev, Be’er Sheva, Israel; 3 The National Institute for Biotechnology in the Negev (NIBN), Ben-Gurion University of the Negev, Be’er Sheva, Israel; St Jude Children's Research Hospital, UNITED STATES

## Abstract

The ε-amino group of lysine residues may be mono-, di- or tri-methylated by protein lysine methyltransferases. In the past few years it has been highly considered that methylation of both histone and non-histone proteins has fundamental role in development and progression of various human diseases. Thus, the establishment of tools to study lysine methylation that will distinguish between the different states of methylation is required to elucidate their cellular functions. The 3X malignant brain tumor domain (3XMBT) repeats of the Lethal(3)malignant brain tumor-like protein 1 (L3MBTL1) have been utilized in the past as an affinity reagent for the identification of mono- and di-methylated lysine residues on individual proteins and on a proteomic scale. Here, we have utilized the 3XMBT domain to develop an enzyme-linked immunosorbent assay (ELISA) that allows the high-throughput detection of 3XMBT binding to methylated lysines. We demonstrated that this system allows the detection of methylated peptides, methylated proteins and PKMT activity on both peptides and proteins. We also optimized the assay to detect 3XMBT binding in crude *E*. *coli* lysates which facilitated the high throughput screening of 3XMBT mutant libraries. We have utilized protein engineering tools and generated a double site saturation 3XMBT library of residues 361 and 411 that were shown before to be important for binding mono and di-methylated substrates and identified variants that can exclusively recognize only di-methylated peptides. Together, our results demonstrate a powerful new approach that will contribute to deeper understanding of lysine methylation biology and that can be utilized for the engineering of domains for specific binders of other post-translational modifications.

## Introduction

Post-translational modification (PTM) of proteins regulates cellular functions and protein activity, forming a separate regulatory network alongside traditional genomic, transcriptomic or translational regulation mechanisms [1,2]. These reversible modifications are controlled by enzymes that add or remove modifications from protein residues. Lysine methylation is a type of PTM that is characterized by the addition of methyl (CH_3_) groups to a lysine residue. S-adenosyl-L-methionine (AdoMet) is the methyl donor during the methylation reaction and is the second most widely used enzyme substrate following ATP [3]. A lysine residue may become mono, di or tri-methylated, leading to increased hydrophobicity and van der Waals interactions with surrounding residues [4].

Lysine methylation occurs on both histone and non-histone proteins and is catalyzed by protein lysine (K) methyltransferases (PKMTs). There are ~50 known members of this family, of which all but one contain a conserved SET domain that carries out the enzymatic activity. In the past decade, both histone and non-histone proteins were shown to be subjected to lysine methylation highlighting the importance of this PTM for the regulation of many different biological processes [4–8]. Thus, PKMTs are considered to be important targets for the development of inhibitors and activators for therapeutic applications.

The three existing states of protein methylation (me1, me2 and me3) extend the diversity of this type of modification. The complexity of lysine modification requires the development of different experimental approaches to identify methylated proteins and to distinguish between the different states of methylation. The use of tritium-labeled SAM has proven efficient for the identification of new methylation events; however, the degree of methylation cannot be determined using this approach. In addition, this method does not allow determining the type of methylated residue, as in addition to lysine, methylation is also observed on arginine, histidine, aspartate and glutamate residues [9,10]. Pan-methyl antibodies that presumably recognize all states of methylation are the most widely used approach to identify methylated proteins in cells. However, they suffer from cross-reactivity and cannot efficiently discriminate between different states of methylation [11].

Proteomic approaches for identification of new methylated substrates include proto-arrays, pull-down and mass-spectrometry methods. New approaches that are based on known methyllysine-binding protein domain were recently reported. For example, HP1-beta chromo-domain [12] was used as a bait against cell extract to identify new methylated substrates. In addition, the 3XMBT domain of L3MBTL1 was employed as an affinity reagent capable of binding to a wide range of mono and di-methylated lysines to identify methylated proteins in the cell [13–15].

Here, we have utilized the 3XMBT domain to develop an enzyme-linked immunosorbent assay (ELISA) that allows the high-throughput detection of 3XMBT binding to methylated lysines. We demonstrated that this approach can be used to detect methylated peptides, methylated proteins and PKMT activity on both peptides and proteins. We have utilized this approach for the engineering of 3XMBT mutants that are highly specific to di-methylated lysine residues.

## Materials and Methods

### Protein expression vectors

pGEX-6p1-MBT and pGEX-6p1-MBT_D355N were kindly provided by Or Gozani's lab. pMAL-MBT, pGEX-6p1-RelA, pMAL-RelA, pMal-SETD7 were previously described in Duchin et al [16]. pGEX-6p1-MBT-T411L mutant was generated by site directed mutagenesis using primers listed in S1 Table as described in Levy et al [17].

### Peptides

Synthetic peptides (un-methylated or methylated) were ordered from GL Biochem or PEPTRON, INC (see peptides sequence in S1 Table). The H3K9, H3K23 and H4K20 unmodified and modified peptides listed in S1 Table were kindly provided by Or Gozani.

### 3XMBT expression and purification

*E*. *coli* cells transformed with pGEX-6p1-MBT were grown at 37°C to OD_600_ of ~0.6 and then induced with 0.2 mM IPTG for four hours at 30°C. The cultures were then centrifuged, re-suspended in lysis buffer (PBS + 0.1% protease inhibitor cocktail (Sigma-Aldrich) + 0.05% benzonase nuclease (Sigma-Aldrich) on ice and lysed by sonication on ice. The lysates were then centrifuged at 4000 rpm in 4°C for 30 minutes. Cleared lysates were loaded onto glutathione sepharose beads (Novagen), washed thoroughly with PBS and eluted with PBS + 10 mg/ml reduced glutathione (Sigma-Aldrich).

### 3XMBT double site saturation mutagenesis

To perform double site saturation mutagenesis on positions 361 and 411 on 3XL3MBTL1, a short fragment of the MBT gene containing these two amino acid positions was amplified by PCR using a mix of primers: fr-MBTL361-NNS, and rev-MBTT411-NNS, with the pGEX-6p1-MBT plasmid serving as a template. The two overlapping sequences of the MBT gene were amplified from the same plasmid in separate reactions, using the primer sets: pGEX-5' with rev-MBT361-overlap and fr-MBT411-overlap with rev-pGEX-3prime. Subsequently, the three fragments were fused together in an assembly PCR reaction, and amplified using the primer set fr-AscI-FLAG with rev-pGEX-3prime in a subsequent PCR reaction (see primers sequence in S1 Table). The entire 3XMBT gene library was digested with BamHI-HF and EcoRI-HF (NEB) for three hours at 37°C and ligated into a pGEX-6p1 vector. Ligation products were transformed into *E*. *coli* strain DH-5α and plated on LB-agar+100 μg/ml ampicillin, and the cloning was validated by sequencing of four randomly selected bacterial colonies. The rest of the bacterial colonies were harvested for plasmid library purification.

*In-vitro* methylation assay using SETD7: Methylation reactions contained 40 μl of crude lysate (expressing the substrate), 5 μM SETD7methyltransferase and 300 μM SAM. A final volume of 50 μl was reached using PBS. The samples were incubated at room temperature for one hour. Before being added to the ELISA assay (see below), the samples were diluted to a final volume of 100 μl using PBS + 0.1% of Tween 20 (PBST) supplemented with 2% BSA.

### High-throughput ELISA detection of peptides and full-length proteins lysine methylation

Each 96-well plate (Greiner, catalog number 655061) was coated with streptavidin (Pierce) at a concentration of 1 μg/ml in PBS and incubated for 1 hour at room temperature. The plate was washed three times with PBST followed by the addition of biotinylated peptide at a concentration of 10 μM in PBS and incubated for one hour at room temperature. The plate was then washed three times with PBST and incubated with PBS+ 3% BSA (PBS-BSA) for one hour at room temperature for blocking. Following additional washing the purified MBT protein or cleared *E*. *coli* cell lysate containing overexpressed MBT protein was then added to the plate at a concentration of 0.02 mg/ml for the purified protein or 1:5 cleared lysate suspended in PBST containing 1.5 mM of DTT (Dithiothreitol) and incubated for one hour at room temperature. The plate was washed three times with PBST and mouse anti-FLAG M2 (Sigma-Aldrich) antibody was then added to the plate at a dilution of 1:5000 in PBS-BSA and incubated for one hour at room temperature. The plate was then washed three times in PBST and incubated with (goat) anti-mouse-HRP conjugated antibody (Santa Cruz) diluted 1:4000 in PBS-BSA for one hour at room temperature followed by five washes with PBST. TMB substrate+chromogen (Dako) was then added to the plate and incubated for 2 minutes at room temperature. Reactions were terminated by the addition of 1N H_2_SO_4_ followed by monitoring at 450nm using a TECAN Infinite M200 plate reader. For detection of full-length methylated proteins, samples were subjected to *in-vitro* methylation assay (see above) before adding them to the GST-coated ELISA plate. To avoid GST dimerization, MBP-MBT (maltose binding protein) was used to detect methylated proteins.

### Mutant protein library expression

100 ng of the pGEX-6p1-MBT 361/411 double site saturation mutant plasmid library were transformed into *E*. *coli* BL21 (DE3) and plated on LB-agar + 100 μg/ml of ampicillin. Individual bacterial colonies, representing individual MBT mutant clones, were picked from the plates, inoculated into 800 μl of LB containing 100 μg/ml ampicillin in a sterile 96-deep well plate (Masterblock, Greiner Bio-One) and grown overnight at 37°C with agitation. The overnight cultures from the deep well plate were inoculated 1:20 into 800 μl of fresh LB containing 100 μg/ml of ampicillin in a new 96-deep well plate, grown for 2.5 hours at 37°C and induced with 0.5 mM IPTG for an additional 4 hours at 30°C. The plates were then centrifuged at 4000 RPM for 15 minutes, the medium discarded and the bacteria pellets frozen at -80°C. The bacterial pellets were then thawed, lysed using BugBuster (Novagen) at room temperature for 30 minutes and then diluted 1:5 with MBT buffer (PBST 0.2% + 1.5mM DTT). The lysates were then cleared by centrifugation at 4000 RPM for 30 minutes. Cleared *E*. *coli* lysates containing overexpressed MBT protein were subsequently loaded onto the high throughput ELISA plate for lysine methylation detection. The catalogued clones from the initial plate were frozen at -80°C in 25% glycerol.

### Structure modeling

The PDB coordinates and the electron density maps were automatically generated in Coot by a direct downloading from the Electron Density Server (http://eds.bmc.uu.se/eds/). The human SCML2 MBT repeats in a complex with histone H2A peptide (monomethyl lysine, pdbID-4EDU)[18] structure was modified according to its electron density or mutated using Coot [19]. The structure of the Lethal(3)malignant brain tumor-like protein 1 3-MBT repeats (dimethyl lysine analog, PDBID-1OYX) [20] was overlapped on 4EDU using the least square fit in Coot to allow the correct orientation of the dimethyl lysine. Figures were prepared with PyMOL (The PyMOL Molecular Graphics System, Version 1.8 Schrödinger, LLC.).

## Results

### ELISA for detection of methylated peptides

**Fig 1:**
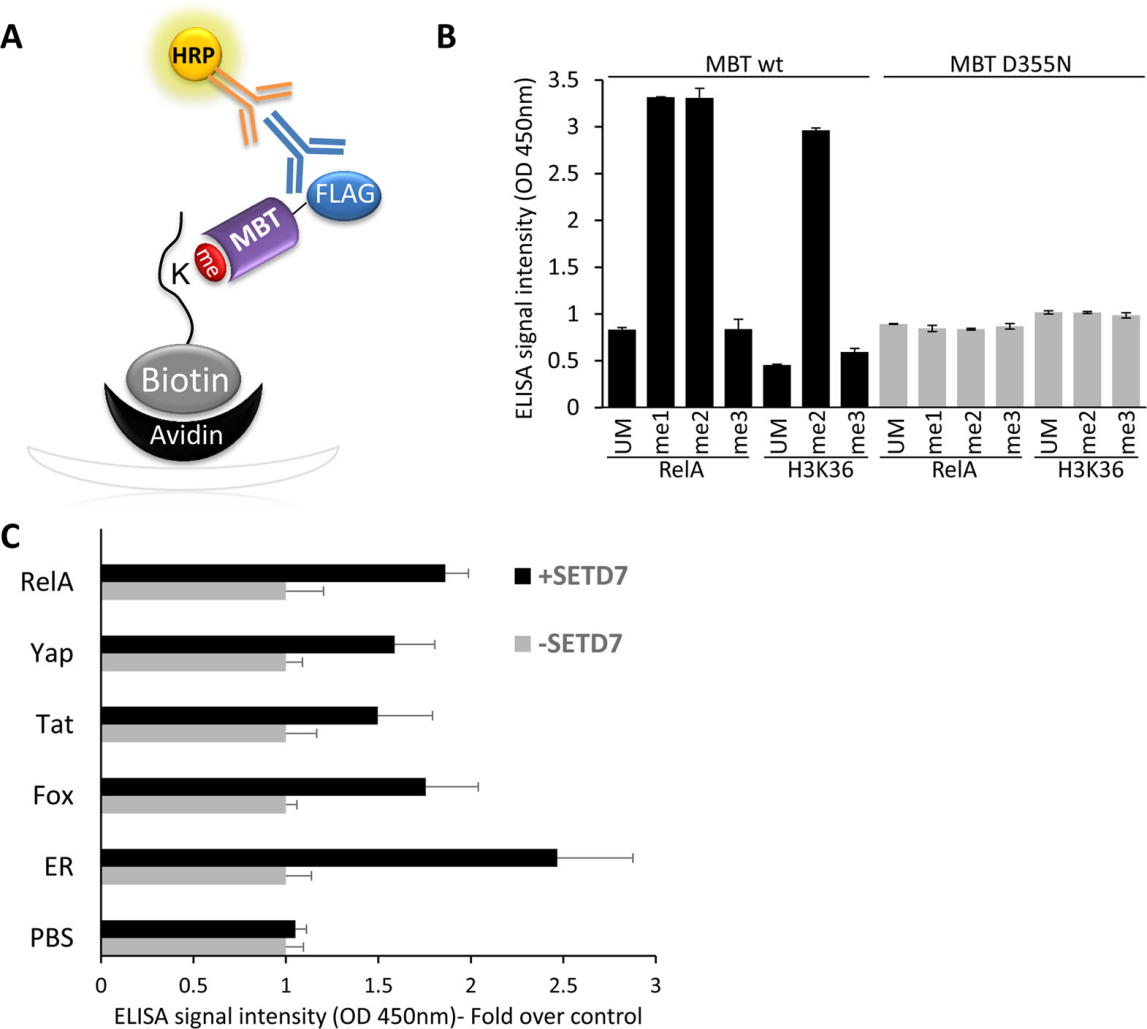
**ELISA-based assay for detection of methylated peptides (A)** Schematic representation of the ELISA. An ELISA plate well is coated with streptavidin and biotinylated methylated peptide. GST-3XMBT domain binds to immobilized methylated peptides, and the ELISA binding signal is measured by subsequent incubation with an anti-GST primary antibody and an HRP-conjugated secondary antibody. **(B)** ELISA signal intensity obtained with the WT and D355N 3XMBT binding to the indicated peptides. **(C)** ELISA for the measurement of lysine methylation of five different peptide substrates following SETD7 *in-vitro* methylation reaction (black bars) or in the absence of SETD7 (grey bars). Data (B+C) are representative of three independent experiments (error bars, S.D.).

To develop an ELISA for the detection of methylated peptides/proteins we utilized purified Flag-GST-3XMBT as a methyl lysine binder (**Fig 1A**). We first tested the ability of the assay to detect differences in the methylation state of a RelA peptide (300–320) and H3K36 peptide–both un-methylated, mono-, di- and tri-methylated [17,21]. To this end, we tested the binding of WT 3XMBT and the D355N inactive mutant to these peptides (**Fig 1B**). As expected [14], we found that the WT 3XMBT domain bound mono- and di-methylated peptides, obtaining high ELISA binding signal relative to the signal for un-methylated or tri-methylated peptides. In contrast, the inactive 3XMBT D355N mutant exhibited low binding to all peptides, consistent with previous observations [14]. We next tested the ability of the assay to monitor the activity of the mono-methyl transferase SETD7 [22–24]. We chose six known SETD7 peptide substrates and subjected them to *in-vitro* methylation reactions utilizing purified SETD7 [25–29]. Following *in-vitro* methylation, the methylation levels of the six peptides were examined by ELISA relative to control reactions containing no enzyme. As expected, the binding signal of the peptides methylated by SETD7 was significantly higher than the background reaction for all peptides (**Fig 1C**). These experiments validated the ELISA method using the 3XMBT domain to detect mono and di-methylated peptides and demonstrated that it can also be used to monitor PKMT activity on specific peptides.

### ELISA for detection of methylated full-length proteins

**Fig 2:**
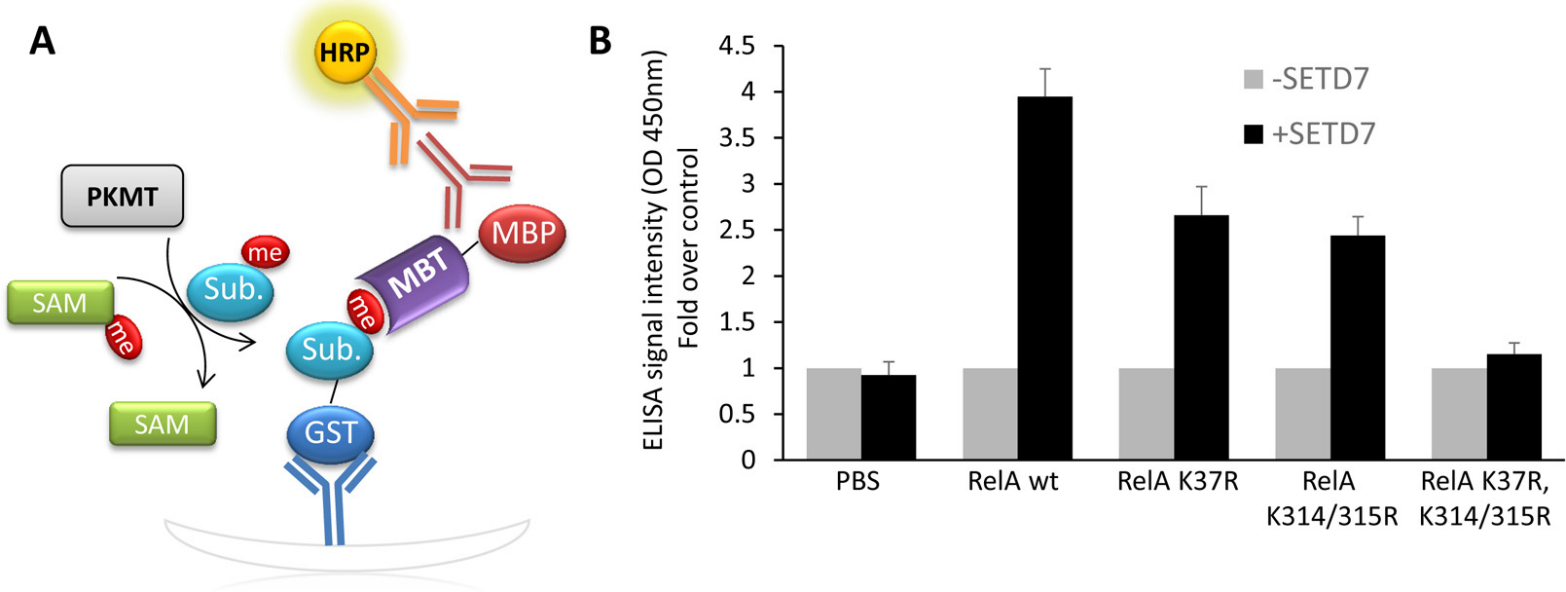
**ELISA for the detection of methylated proteins (A)** Schematics of the ELISA for the identification of methylated proteins (see text for details). **(B)** ELISA signal intensity of WT GST-RelA and the K37R, K314/315R and K37R/K314/315R following *in-vitro* methylation reaction in the absence (grey bars) or presence (black bars) of recombinant SETD7. Following the reaction, the degree of methylation was determined by MBP-MBT fusion protein as described above. PBS served as a negative control for the reaction. Data are representative of three independent experiments (error bars, S.D.).

To assess the ability of the ELISA to detect methylated lysines on full-length proteins (**Fig 2A**), we examined RelA methylation by SETD7. SETD7 was recently shown to methylate RelA on 3 different lysines: K37, K314 and K315 [25,26]. Thus, we subjected the WT RelA (1–431) and the K37R, K314R/K315R and K37R/K314R/K315R mutants to *in-vitro* methylation by SETD7 and utilized the ELISA method to detect RelA methylation. To avoid GST dimerization between the recombinant RelA and the 3XMBT, we utilized a MBP (maltose binding protein) MBT fusion protein. We observed higher ELISA binding signal for the WT RelA relative to the signal detected for the K37R and K314R/K315R mutants, consistent with the decrease in available methylation sites in these mutant proteins. The signal measured for the triple mutant signal that is devoid of SETD7 methylation sites was similar to the control signal without enzyme, providing further support for the utility of the assay to detect protein methylation (**Fig 2B**). Taken together, these results indicate that the ELISA can be used for the detection of protein methylation and is sensitive for the detection of methylation on distinct sites of protein substrates.

### Detection of methylated lysine in bacterial lysates

**Fig 3:**
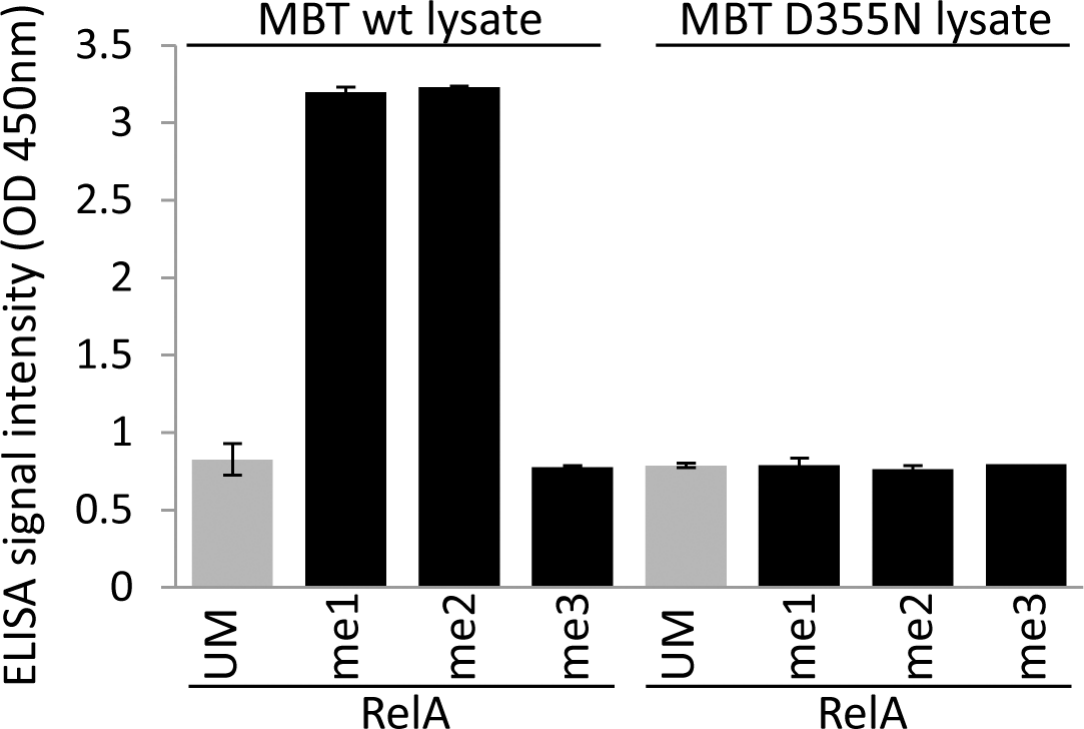
Detection of methylated lysine by 3XMBT bacterial lysates. ELISA signal intensity following incubation of bacterial lysates obtained from *E*. *coli* cells overexpressing WT or D355N mutant 3XMBT with RelA methylated (black) and un-methylated (grey) peptides. Data are representative of three independent experiments (error bars, S.D.).

Having demonstrated that the MBT ELISA assay can be utilized for the specific identification of mono and di-methylated peptides and proteins using purified MBT proteins, we next tested whether this assay can be used for the detection of peptide methylation in crude bacterial lysates. This method simplifies the high-throughput screening of MBT mutant libraries for the identification of mutants with novel binding specificity (see below). As these libraries can include hundreds or even thousands of mutants, purification of each MBT mutant prior assaying is not feasible. To examine the utility of the assay to monitor methylation in crude cell extracts, bacterial cell lysates overexpressing 3XMBT were subjected to ELISA with methylated RelA peptides (**Fig 3**). As observed with the purified 3XMBT, we obtained high ELISA binding signal for mono and di-methylated peptides, but low background signals for the un-methylated and tri-methylated peptides. Consistently, lysates obtained from *E*. *coli* cells expressing the MBT D355N mutant exhibited low binding signals for all peptides (**Fig 3**). These experiments demonstrate the utility of the assay to detect 3XMBT binding to methylated peptides in crude *E*. *coli* lysates and pave the way for the screening of 3XMBT mutant libraries.

### Engineering of 3XMBT for high specificity for di-methylated peptides

**Fig 4:**
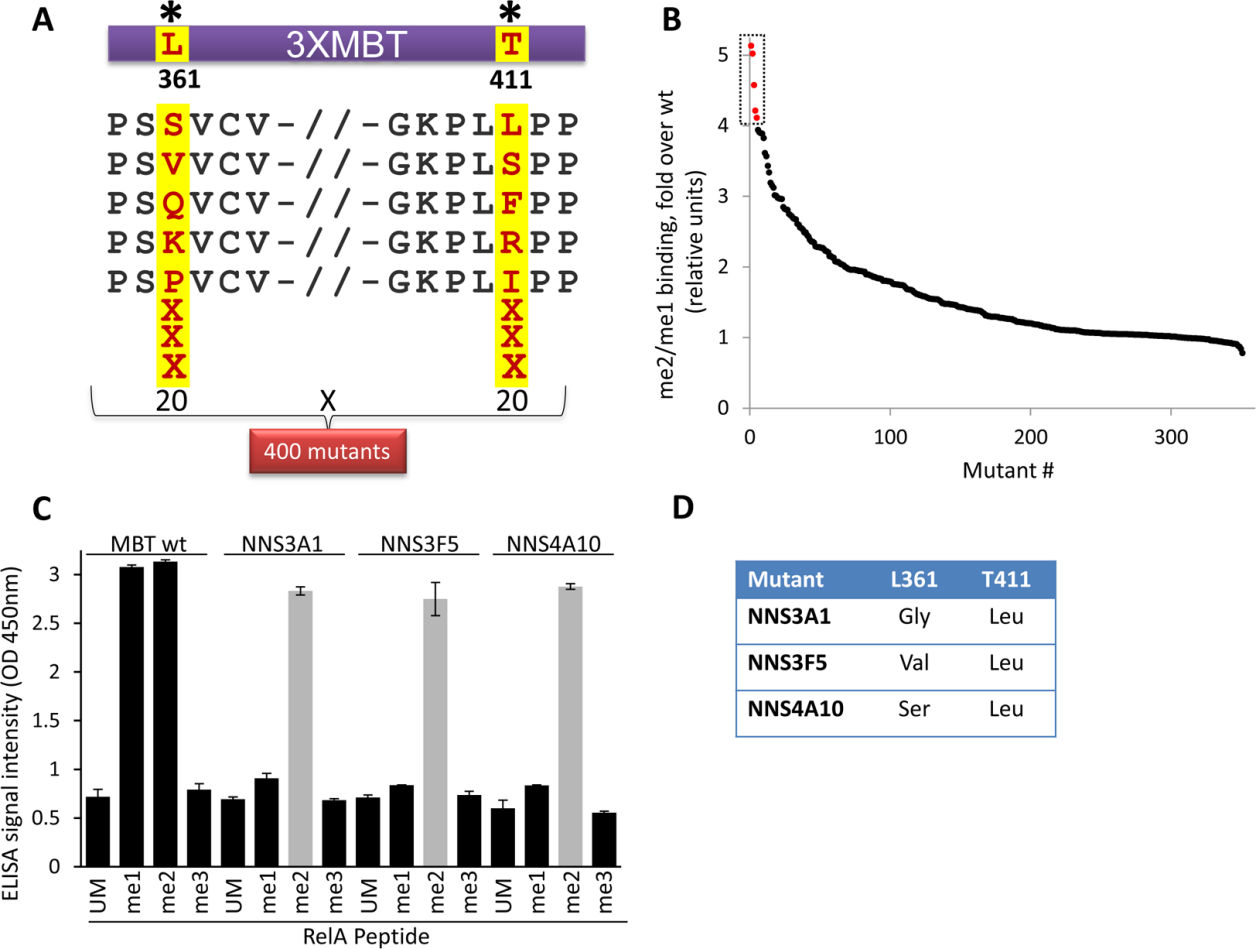
**Engineering of 3XMBT with high specificity for di-methyllysine (A)** Schematic representation of mutation sites on the 3XMBT gene. Mutagenic primers with degenerative codons for L361 and T411 were employed to generate a double saturation mutant library. Each position can be substituted to all of the 20 naturally-occurring amino acids generating a library of 400 possible mutant proteins. Highlighted mutants at positions 361 and 411 are random examples of possible substitutions. **(B)** Screening of ~360 3XMBT mutant library for binding to di-methylated and mono-methylated RelA peptides. Results are shown as fold increase of me2/me1 signal relative to the WT 3XMBT. Highlighted are clones chosen for characterization using a panel of RelA peptides. **(C)** Characterization of selected 3XMBT mutants (NNS3A1, NNS3F5 and NNS4A10) binding to the full spectrum of RelA methylated peptides. Bacterial lysates expressing the different clones were used in these experiments. **(D)** The mutations in positions 361 and 411 found in the 3XMBT mutants exhibiting high specificity for di-methylated lysine. Data in C are representative of three independent experiments (error bars, S.D.).

As demonstrated here (**Figs 1 and 3**) and in previous studies, the 3XMBT domain binds both mono and di-methylated peptides with similar affinities [18]. This lack of MBT specificity hinders the identification of specific protein methylation states *in-vitro* and in cells. Thus, we developed a directed evolution approach for the engineering of 3XMBT mutants with high specificity to one specific methylation state. Previously, residues 361 and 411 in the L3MBTL1 were shown to be important for binding to mono and di-methylated substrates. It was shown that point mutations in these positions lead to an altered binding affinity ratio between mono and di-methylated substrates [18]. Thus, we generated a 3XMBT mutant library by double site saturation mutagenesis of residues 361 and 411. The highlighted mutants at positions 361 and 411 are random examples of possible substitutions (**Fig 4A**). Each of the 400 3XMBT mutants was overexpressed in *E*. *coli* and individually tested for binding to mono and di-methylated RelA peptides (**Fig 4B and S2 Table**). As expected, we found that most mutants exhibited similar or lower binding activity compared to the WT protein. However, some mutants exhibited significant binding only to the di-methylated peptide. Mutants exhibiting more than a 3.5-fold increase in binding signal for the di-methylated peptide over the mono-methylated peptide relative to the WT protein were selected for further analysis. Of these, we next examined clones NNS3A1, NNS3F5, and NNS4A10 for binding to the full spectrum of methylated RelA peptides including the mono, di and tri-methylated peptides. Using both cell lysates and purified recombinant proteins **(Fig 4C and S1 Fig, respectively)**, we found that all clones can specifically bind only the di-methylated RelA peptide, demonstrating our ability to engineer a 3XMBT that is highly specific for one methylation state. Strikingly, sequence analysis of these mutants revealed that, while position 361 could be substituted with different amino acids, position 411 was mutated to leucine in all variants, suggesting that L411 confers high binding specificity for di-methylated lysine (**Fig 4D**).

**Fig 5:**
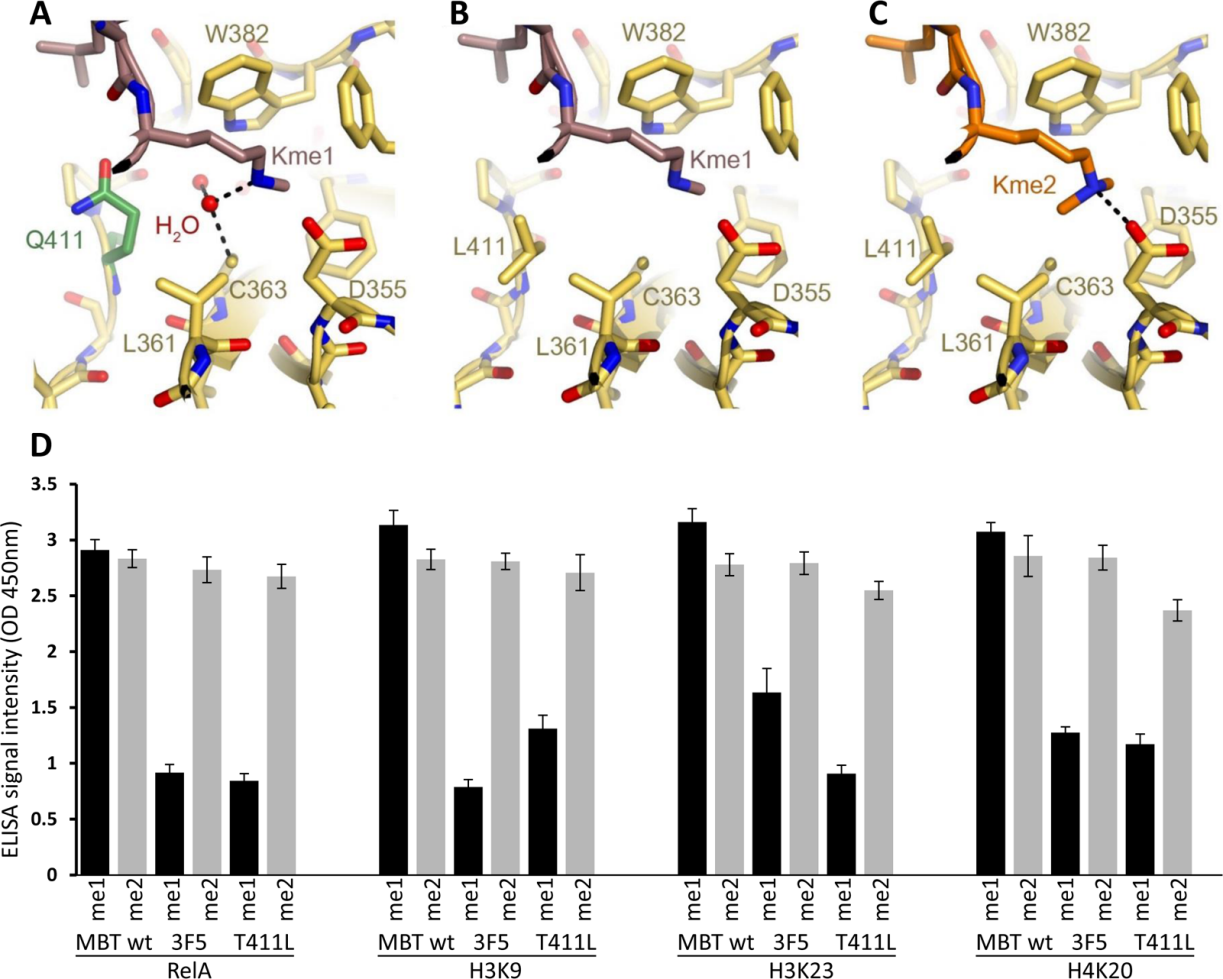
**Structural basis for the high specificity of T411L 3XMBT mutant toward di-methyllysine (A)** The binding pocket of WT MBT domain bound to mono-methylated lysine. The mono-methylated lysine is stabilized by hydrogen bonds with a surrounding water molecule and polar residues. **(B)** An *in silico* model of MBT T411L mutant binding to mono-methylated lysine. The presence of leucine at position 411 leads to increased hydrophobicity of the pocket and the possible exclusion of the water molecule that is important for stabilizing the mono-methylated lysine. (**C**) An *in silico* model of MBT T411L mutant binding to di-methylated lysine. This mutant binding pocket is more hydrophobic enabling the stabilization of the di-methylated peptide. **(D)** Characterization of NNS3F5 (3F5) and the T411L recombinant protein mutants binding to the indicated methylated peptides. Data in D are representative of two independent experiments (error bars, S.D.). PDB identifier for A-C is 1OYX.

To understand the structural basis for the novel specificity of the engineered MBT mutants and rationalize how the T411L mutation can lead to the exclusion of mono-methylated lysine from the 3XMBT binding site, we have analyzed the crystal structure of the WT 3XMBT bound to mono-methylated lysine and di-methylated lysine analog ligands **(Fig 5A–5C)**. In addition, we introduced the T411L mutation *in silico* within 4EDU structure to examine its possible effects on the 3XMBT binding to the mono and di-methylated lysine. Since the binding site of the 3XMBT in the crystal structure lacks methylated lysine, we performed the analysis on the structure of 4EDU and the residues in the 3XMBT were assigned following structural alignment between the two crystal structures (4EDU and 1OYX, **S2A Fig**). Careful analysis of the electron density map of the 3XMBT structure revealed the presence of a water molecule in the binding site that is located in close vicinity to the methylated lysine and the inversion of the methyl group of the mono-methylated lysine (**S2B Fig)**. Analysis of the mono-methylated lysine located within the WT 3XMBT binding site reveals that the methyl-ammonium group is stabilized by a network of hydrogen bonds mediated by the water molecules (**Fig 5A**). Analysis of the binding site of the T411L mutant reveals that the mutation to leucine can significantly increase the hydrophobicity of the binding site, leading to an exclusion of the water molecule and consequently to disfavored binding to mono-methylated lysine (**Fig 5B**). However, the increase in size and hydrophobicity of the di-methylated lysine can stabilize its interactions with the hydrophobic environment of the mutated binding site (**Fig 5C**). Based on the structural model we hypothesized that the leucine substitution mutant at T411 may be sufficient to increase the selectivity toward di-methyl lysines. To this end, the binding specificity to different peptides of recombinant T411L 3XMBT mutant was compared to the WT and the NNS3F5 recombinant 3XMBT proteins **(Fig 5D and S3 Fig)**. As expected, WT 3XMBT recognizes mono and di-methyl peptides. Strikingly, the binding properties toward the different di-methyl peptides (RelA, H3K9, H3K23 and H4K20) of the T411L was similar to the NNS3F5 clone which is mutated in both L361 and T411 (L361V and T411L, respectively). Taken together, our data supports a model by which these structural changes can lead to a decreased affinity to mono-methylated lysine while maintaining affinity to the di-methylated lysine.

## Discussion

In this paper we have described the development of a high-throughput ELISA to detect methylated lysines on peptides and globular proteins. We demonstrated the utility of this assay to examine PKMT enzymatic activity on peptide and protein substrates and show the sensitivity of the assay for the detection of different methylation sites on RelA. We have optimized the assay to detect MBT binding in crude *E*. *coli* lysates to enable the high throughput screening of MBT mutant libraries. Finally, we have utilized this assay to engineer MBT variants that are highly specific to di-methylated lysine and obtained new insights into MBT-methyllysine interactions.

The emerging importance of lysine methylation in regulating many biological processes has led many research groups to develop tools to identify new methylation events [13–15,30–32]. Our ELISA-based screening approach and the engineering of a novel 3XMBT domain that can specifically recognize di-methyllysine over mono-methylated lysine complements the existing tools for the identification of methylated proteins. The combination of our engineered MBT variant together with other biochemical and mass-spectrometry based approaches [33–35] can serve as a powerful tool to investigate the specific abundance of di-methyllysine in proteins and may be used to determine the functional importance of this modification to cellular processes. We envision that further engineering of methylated lysine binding domains using the ELISA approach can lead to a repertoire of domains capable of specific recognition of mono-methylated, di-methylated or tri-methylated proteins in the cell. Finally, we believe that this approach can be utilized for the engineering of domains for the specific binding of other post translational modifications involved in diverse and essential biological processes in living cells.

## Supporting Information

S1 Fig **Detection of methylated RelA peptides by 3XMBT recombinant protein mutants clones (A)** Coomassie stain of the recombinant proteins used in Fig 5D and S3 Fig. **(B)** ELISA signal intensity following incubation of WT, NNS3A1, NNS3F5 or NNS4A10 mutants 3XMBT purified proteins with the indicated RelA peptides. Data (B) are representative of three independent experiments (error bars, S.D.).
(TIF)

S2 Fig **Analysis of the MBT monomethyl lysine binding site (A)** Structural overlap of 1OYX methylated lysine binding site (represented as yellow sticks) on 4EDU (represented as green sticks). Carbon, Oxygen and Nitrogen atoms are Yellow (1OYX) or Green (4EDU), red and blue respectively. **(B)** The electron density map (represented as thin mesh, in blue 1σ 2Fo-Fc map and in green positive 3σ Fo-Fc map as generated by Coot) and the modified structure of 4EDU (presented as lines, green, red, blue and light green represent Carbon, Oxygen, Nitrogen and Sulphur atoms, respectively), indicate for the missing water molecule (red stars).
(TIF)

S3 Fig Binding preferences of purified T411L and NNS3F5 clones to di-methyl peptides Characterization of NNS3F5 and the T411L mutants binding to the indicated methylated peptides. Data are representative of two independent experiments (error bars, S.D.).
(TIF)

S1 Table Primers and peptides used in the study. (XLSX)

S2 Table Raw data for Fig 4B. (XLSX)
