# Supplementary figures and images for: Engineering of Methylation State Specific 3xMBT Domain Using ELISA Screening
Source: PLoS One. 2016 Apr 25;11(4):e0154207. doi: 10.1371/journal.pone.0154207 (PMC4844143; doi:10.1371/journal.pone.0154207)

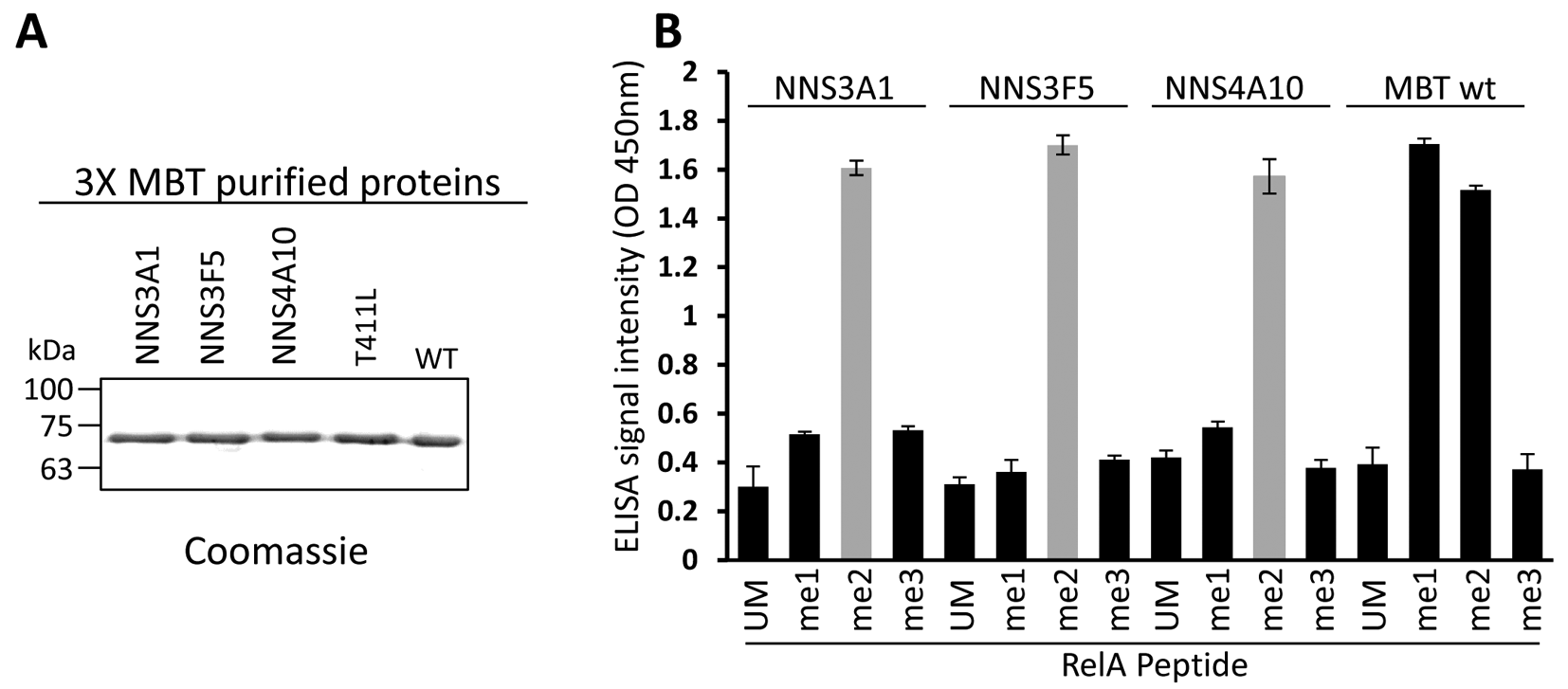

Supplement: S1 Fig — Detection of methylated RelA peptides by 3XMBT recombinant protein mutants clones (A) Coomassie stain of the recombinant proteins used in Fig 5D and S3 Fig. (B) ELISA signal intensity following incubation of WT, NNS3A1, NNS3F5 or NNS4A10 mutants 3XMBT purified proteins with the indicated RelA peptides. Data (B) are representative of three independent experiments (error bars, S.D.). (TIF) [file pone.0154207.s001.tif]

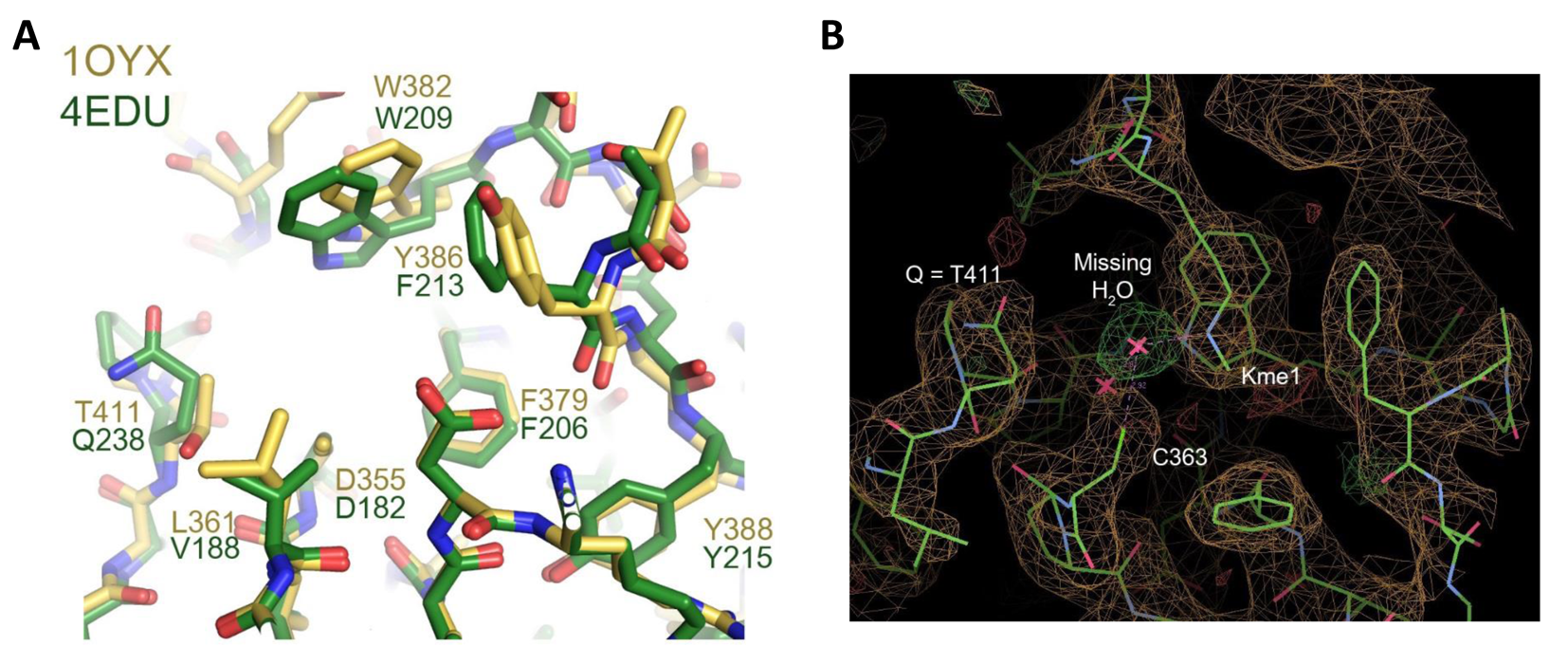

Supplement: S2 Fig — Analysis of the MBT monomethyl lysine binding site (A) Structural overlap of 1OYX methylated lysine binding site (represented as yellow sticks) on 4EDU (represented as green sticks). Carbon, Oxygen and Nitrogen atoms are Yellow (1OYX) or Green (4EDU), red and blue respectively. (B) The electron density map (represented as thin mesh, in blue 1σ 2Fo-Fc map and in green positive 3σ Fo-Fc map as generated by Coot) and the modified structure of 4EDU (presented as lines, green, red, blue and light green represent Carbon, Oxygen, Nitrogen and Sulphur atoms, respectively), indicate for the missing water molecule (red stars). (TIF) [file pone.0154207.s002.tif]

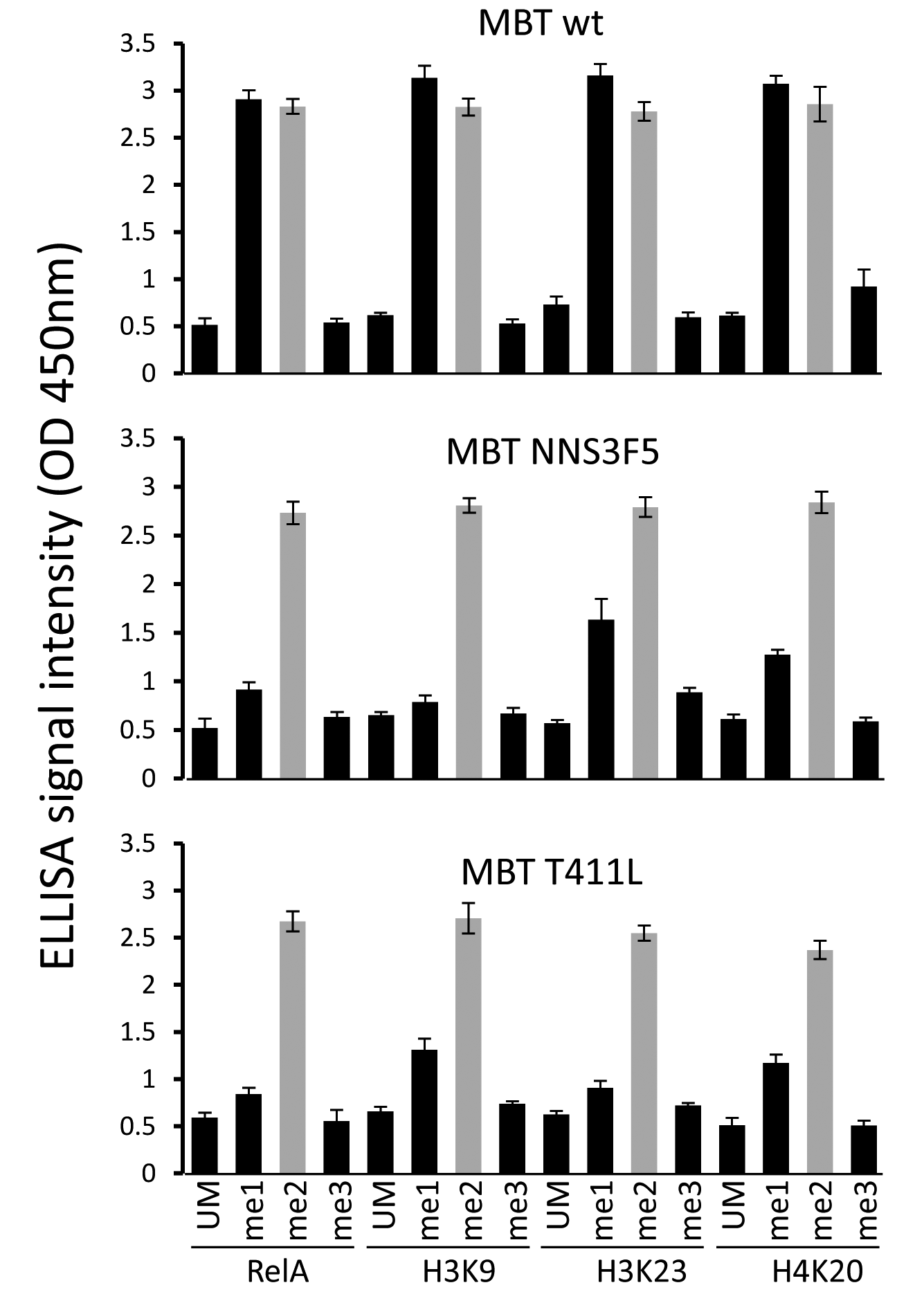

Supplement: S3 Fig — Data are representative of two independent experiments (error bars, S.D.). (TIF) [file pone.0154207.s003.tif]
